# Use of Analgesics and Sedatives in Nontraumatic Patients after Sudden Cardiac Arrest during the Peri-Resuscitation Period in the Emergency Department

**DOI:** 10.3390/jcm13154563

**Published:** 2024-08-05

**Authors:** Sebastian Dąbrowski, Lucyna Tomaszek, Andrzej Basiński

**Affiliations:** 1 Department of Medical Rescue, Faculty of Health Sciences with the Institute of Maritime and Tropical Medicine, Medical University of Gdańsk, 80-210 Gdańsk, Poland; andrzej.basinski@gumed.edu.pl; 2 Department of Specialist Nursing, Faculty of Medicine and Health Sciences, Andrzej Frycz Modrzewski Krakow University, 30-705 Kraków, Poland; ltomaszek@igrabka.edu.pl; 3 Department of Thoracic Surgery, Institute of Tuberculosis and Lung Diseases, 34-700 Rabka-Zdrój, Poland

**Keywords:** analgesia, sedation, sudden cardiac arrest, resuscitation, emergency department

## Abstract

**Background:** While cardiopulmonary resuscitation (CPR) may be life-saving for patients in cardiac arrest, it simultaneously puts them at risk for skeletal and soft tissue injuries. The prevalence of cardiovascular and thoracic wall injuries related to CPR varies significantly in the literature, from 21% to more than 78%. After restoration of circulation, ischemia–reperfusion brain injury ensues. Sedation is one of the interventions that can reduce its effects on brain cells. The purpose of this study was to analyse the use of analgesics and sedatives in nontraumatic patients after sudden cardiac arrest in the peri-resuscitation period in the emergency department. **Methods:** This was a retrospective cohort study. An analysis was performed on the electronic health records of 131 patients who underwent CPR, with ages ranging from 24 to 96 years. The study protocol was in accordance with the Declaration of Helsinki. **Results:** Chest wall injuries were assessed based on the results of X-ray imaging (*n* = 39; 31%) and computed tomography (*n* = 87; 69%). Of the 126 cases, 17.5% had rib fractures and 6.3% had rib and sternal fractures. Almost 78% of the patients (*n* = 102) received sedatives and/or analgesics during the peri-resuscitation period. Monotherapy was used in 85 cases. Among these drugs the most frequently mentioned were midazolam (45.2%), fentanyl (26.8%), and propofol (20.8%). **Conclusions:** As only two-thirds of the patients received sedation and half received analgesics, there is still room for a broader incorporation of analgesia and sedation into peri-resuscitation care protocols.

## 1. Introduction

In recent years, more than ever, emphasis has been placed on good-quality chest compressions during cardiopulmonary resuscitation. The implementation of mechanical compression devices has allowed us to prolong resuscitative efforts much longer than using only manual chest compressions. Although cardiopulmonary resuscitation (CPR) can save the lives of patients in cardiac arrest, it also puts them at risk for skeletal and soft tissue injuries. The prevalence of cardiovascular and thoracic wall injuries associated with CPR varies significantly in the literature, ranging from 21% to over 78% [1,2]. This diversity can be attributed to numerous factors, such as the effectiveness of chest compressions, the thoroughness in recognizing these complications among both survivors and non-survivors, and the use of different diagnostic techniques in detecting such injuries. Patients subjected to CPR with automated or active compression–decompression devices often exhibit a higher frequency of cardiothoracic injuries [2]. The use of analgesics seems prudent in these cases. Sedation is included in the 2021 revision of the European Resuscitation Council (ERC) guidelines for post-resuscitation care. Along with the already-recommended targeted temperature management, it may play a role in decreasing brain metabolic rates and thus mitigate the effects of ischemia–reperfusion injury [3].

The purpose of this study was to analyse the use of analgesics and sedatives in nontraumatic patients after sudden cardiac arrest in the peri-resuscitation period in the emergency department. An attempt was made to answer the following questions:

1. What was the incidence of chest injuries in patients after CPR?
2. How often were sedatives and analgesics used in patients in the peri-resuscitative period?
3. What factors influenced the use of sedative and/or analgesic drugs during the peri-resuscitation period?

## 2. Materials and Methods

### 2.1. Study Design, Setting, and Ethical Considerations

This was a retrospective cohort study. An analysis of 131 electronic health record data of patients who underwent CPR, with ages varying between 24 and 96 years, was performed. The study protocol was in accordance with the Declaration of Helsinki. This study followed the guidelines of the RECORD statement (The REporting of studies Conducted using Observational Routinely collected health Data) [4].

The Comarch Optimed NXT hospital electronic medical system was used for the analysis. This study was carried out in two emergency departments of municipal hospitals in Gdańsk, which belong to the same healthcare entity.

### 2.2. Participants

The inclusion criteria for the retrospective analysis were as follows: age 18 years or older, underwent CPR, and chest compressions administered manually or with the help of the Lucas device.

Exclusion criteria were age younger than 18, traumatic cardiac arrest, duplicates, and incomplete resuscitation protocol.

### 2.3. Variables

The data obtained included demographic data (age and sex) and clinical data such as cause of SCA, duration of CPR (minutes), CPR techniques (manual or Lucas device), the presence of rib or sternal fractures, assessment of pain and sedation, and administration of sedatives and analgesics.

Sedatives are described as a variety of therapeutic substances that decrease cerebral function and can produce a continuum of cognitive states, from minimal sedation to general anaesthesia. Analgesics have pain relief activity and work on the physiological mechanisms of pain. There are many classes of analgesics, but opioid analgesics, acting through opioid receptors, remain the mainstay of therapy for alleviating pain in critically ill patients. Opioids—in large doses—may induce the state of sedation.

Diagnoses of chest wall injury was made in patients using chest imaging techniques, such as X-rays or computed tomography (CT) scans.

### 2.4. Statistical Analysis

A statistical analysis was performed using STATISTICA v.13 (TIBCO Software Inc., Kraków, Poland, 2017). The Chi-square test and Fisher’s exact test were used to compare categorical data, while the Mann–Whitney U test was used to compare differences between two independent groups for continuous variables. The Spearman’s rank coefficient of correlation (R) describes the relationship between two continuous variables. Categorical variables were recorded as total numbers and percentages, and continuous data were shown as median, lower, and upper quartiles. A *p*-value < 0.05 was considered statistically significant.

## 3. Results

### 3.1. Demographic and Clinical Characteristics of the CPR Cohort

**Figure 1:**
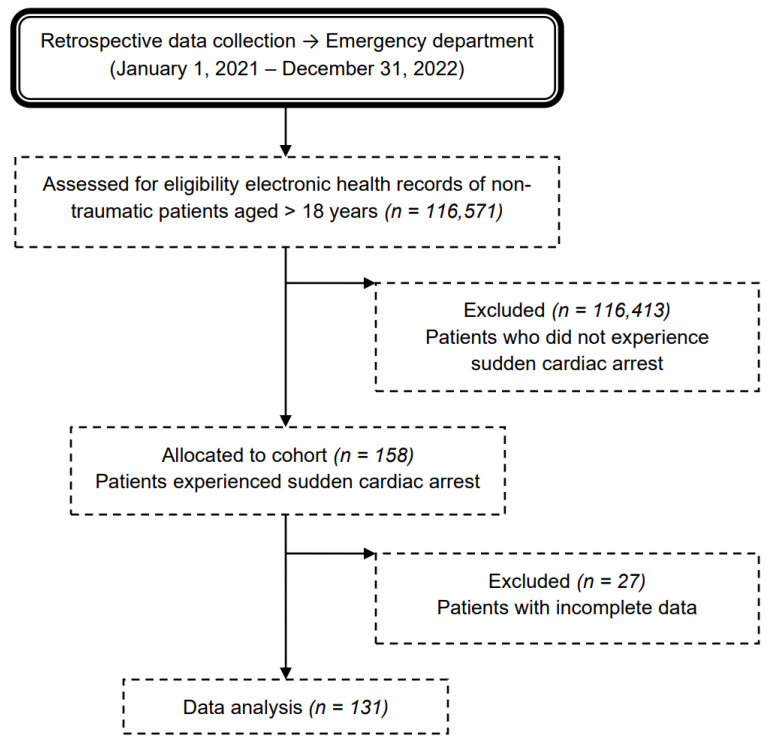
Flow chart of the medical record review process for nontraumatic adult patients admitted to the emergency department due to sudden cardiac arrest.

The final retrospective analysis was performed on 131 patients who experienced SCA. Eighteen cases (12%) were excluded from the analysis due to incomplete data records at the time of CPR (Figure 1 shows a flow chart of the cohort).

The demographic and clinical characteristics of the CPR cases are presented in Table 1. There were 57 women (43.5%) and 74 men (56.5%) in the study group. The median age of the study group was 69 years (mean 67.7 ± 16.5). There was a statistically significant difference between the age of women and men (median 76 [64; 84] vs. 63 [53; 74]; Z = −3.47; *p* = 0005). The youngest two patients were men aged 24 years, and the oldest were two women aged 96 years. The largest age group of the study group consisted of patients aged 60–70 years (22%). The most common causes of SCA were heart failure (30.5%) and myocardial infarction (19.1%), which were primary diagnoses according to ICD-10 at discharge from the emergency department. The distribution of causes of SCA was similar in the group of women and men (χ^2^ = 14.38; *p* = 0.21). The median duration of CPR was 12 min. CPR in the male group was significantly longer than that in the female group (15 [9; 25] vs. 10 [6; 13] min; Z = 3.80; *p* = 0.0001). There was a weak negative correlation between age and duration of CPR (R = −0.27; t = −3.23; *p* = 0.001). Manual chest compressions were applied in 80.1% of the cases (*n* = 105), the Lucas device was used in two patients (1.5%), and a mixed method of resuscitation was performed in the remaining 24 cases (18.3%).

### 3.2. Chest Wall Injuries

**Figure 2:**
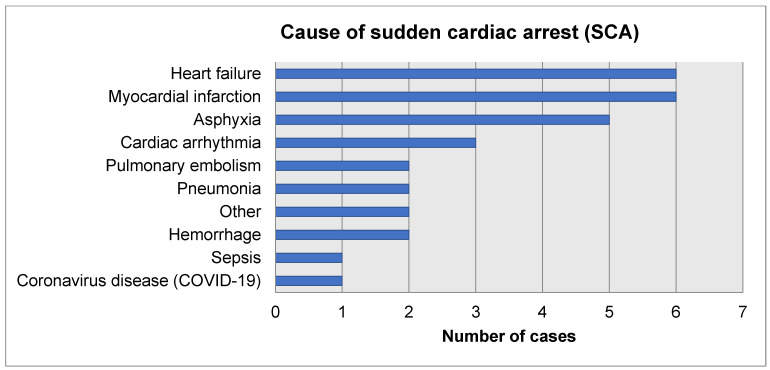
Cause of SCA in the chest wall injury cohort (*n* = 30).

CPR-related chest wall injuries were evaluated based on the results of X-ray imaging (*n* = 39; 31%) and computed tomography (*n* = 87; 69%). Of the 126 cases, 17.5% had rib fractures and 6.3% showed rib and sternal fractures (Table 2). There were no significant differences in the median age of patients with and without fractures (62 [47; 76] vs. 71 [59; 82]; Z = −1.94; *p* = 0.052). More males sustained fractures than females (*n* = 23; 32.4% vs. *n* = 7; 12.7%; χ^2^ = 6.61; *p* = 0.011). The incidence of injuries was similar in the manual and mixed-chest-compression groups (*n* = 21; 21% vs. *n* = 9; 34.6%; χ^2^ = 2.11; *p* = 0.195). The duration of CPR was significantly longer in the fracture group compared to the non-fracture group (median 18 [13; 25] vs. 10 [6; 17] min; Z = 3.38; *p* = 0.0007). The cause of SCA in the chest wall injury cohort is presented in Figure 2.

### 3.3. Pain and Sedation

Almost 78% of the patients (*n* = 102) received sedatives and/or analgesics during the peri-resuscitation period. Monotherapy was used in 85 cases. Among these drugs (Table 3), the most frequently mentioned were midazolam (45.2%), fentanyl (26.8%), and propofol (20.8%). The drugs were administered by continuous infusion, with a few instances of a prior bolus administration. The proportion of patients treated with these medications was similar in the fracture and non-fracture groups (χ^2^ = 1.38; *p* = 0.24). The CPR time was significantly shorter in patients who received sedative and analgesic drugs than in those who did not receive such treatment (median 10 [6; 17] vs. 20 [12; 32] min; Z = −3.37; *p* = 0.0007).

Only in two cases of 73-year-old patients, where the suspicion of chest injury was not confirmed by imaging tests, was a measurement of pain intensity recorded during their stay in the emergency department: once using the NRS scale and the other time using the COPT scale.

## 4. Discussion

Fractures of the ribs and sternum due to chest compressions seem to be a frequent finding reported in adult patients after CPR. The prevalence of cardiovascular and thoracic wall injuries related to CPR varies significantly in the literature, from 21% to more than 78%. This diversity can be attributed to several factors, including the effectiveness of chest compressions, the thoroughness in identifying complications among both survivors and non-survivors, and the use of various diagnostic techniques to detect such injuries. In our study, 31% of the patients had a chest radiograph performed, which showed skeletal damage in 5.4% of the cases. Chest CT was performed in 69% of the patients and showed fractures in the ribs and/or sternum in 32% of the cases. According to some authors, the sensitivity of chest radiographs is at best 50%, especially when using a standard anterior–posterior assessment [5]. In a paper by Lederer et al., post-mortem findings from chest X-rays were in stark contrast to forensic autopsy results in patients after cardiopulmonary resuscitation. Rib fractures were more than twice as common in autopsies compared to chest radiographs, while sternum fractures were almost twice as common [6].

Complete cessation of blood circulation during cardiac arrest leads to anoxia and fast depletion of cerebral oxygen stores and ultimately to a cascade of devastating intracellular derangements [7]. Even with high-quality chest compressions, only a fraction of cardiac output may be achieved [8]. Thus, inevitably, after restoration of circulation, ischemia–reperfusion brain injury ensues. To mitigate its effects on neuronal and glial cells, various interventions are employed with the intention of decreasing the cerebral metabolic rate. One of these interventions is sedation. It is included in the 2021 revision of the European Resuscitation Council (ERC) guidelines for post-resuscitation care by Nolan et al. It might, by ensuring proper mechanical ventilation and avoiding haemodynamic instability, prevent the consequences of post-ROSC brain oedema, which can lead to brain death [3]. Although sedation after ROSC is not uniformly endorsed [9], a recent paper by Takamitsu Ikeda et al. points to the beneficial effects of early sedation in mice after ROSC, e.g., amelioration of histologic brain injury and attenuation of early cerebral hyperaemia [10]. In our study, 74% of all patients received some sedation with midazolam as the predominant drug, followed by propofol. Ketamine, a non-competitive NMDA receptor antagonist with unique analgesic and sedative properties, was used in only two instances. The first instance involved a 53-year-old man resuscitated for 30 min due to bronchospasm and asphyxia from drowning. A CT scan revealed a fracture in the rib and sternum. The second instance involved a 68-year-old patient who suffered cardiac arrest due to shock from gastrointestinal perforation; his trachea was intubated, and he was taken to the operating theatre for surgery. In both cases, ketamine was used only as a bolus to facilitate intubation.

Propofol primarily acts as a GABA_A_ receptor agonist. It is a negative inotropic agent that can cause hypotension both through its negative inotropism and systemic vasodilation. Patients with already-impaired heart function, reduced blood volume, or low vascular tone (such as in sepsis) may be more prone to experiencing significant hypotension after its administration [11]. Midanium is a GABA_A_ agonist and is relatively safe from a cardiovascular perspective, having only mild negative inotropic effects and causing small decreases in blood pressure. Ketamine, the NMDA antagonist, acts on the sympathetic nervous system causing haemodynamic effects, i.e., tachycardia and hypertension, potentially facilitating recovery of systemic blood pressure during CPR. In contrast, in ICU patients with cardiac dysfunction, it can lead to a decline in cardiac index [11]. Interestingly, in a paper by Jung et al., there were no significant changes in mean blood pressure or catecholamine requirements in patients receiving ketamine infusion [12]. Ketamine’s anti-inflammatory actions may attenuate the systemic inflammatory response to tissue injury. By mitigating intracellular calcium buildup, ketamine decreases the generation of reactive oxygen species (ROS) and can prevent the initiation of apoptotic cascades [11].

It should be emphasised that sedatives—except ketamine—do not provide analgesia. Analgesics were administered to almost 43% of all patients. Among these, fentanyl—a short-acting opioid—was administered to almost 81% of the patients and morphine was administered to 16% of the patients. Opioids may also be part of the analgosedation protocol, where, in addition to providing pain relief, they complement the effects of sedative drugs. In this context, they can enhance the haemodynamic effects of sedatives, such as benzodiazepines or propofol, potentially causing hypotension [10]. In our study, almost 39% of patients received analgosedation. Those patients who underwent long-term resuscitation were more likely to have rib and/or sternal fractures, and yet, they were less likely to receive analgesics during their stay in the emergency department. This could be related to their very serious conditions, e.g., profound circulatory failure with hypotension, and fears that this failure should not be aggravated by the supply of sedative drugs and/or strong analgesics. Furthermore, future application of targeted temperature management (TTM) with deep sedation and muscle paralysis will certainly add to the haemodynamic instability of this type of patients [13]. Another reason for this approach could be the belief of the medical personnel that the patient, after long resuscitation, had suffered deep and irreversible anoxemic brain damage, which clearly translates into a poor prognosis for survival and limits further therapy, which is considered futile at this point.

To our knowledge, no sedatives or analgesics were administered during resuscitation efforts by either emergency responders or emergency department personnel. However, in two patients with in-hospital cardiac arrest, ketamine was used for tracheal intubation. Administration of these drugs is not part of the standard resuscitation guidelines; instead, their use depends on the clinical scenario and the experience of the rescuers. With increasing awareness of CPR-induced consciousness—a phenomenon occurring only during chest compressions—some local protocols now advocate for the use of sedatives and analgesics during CPR [14,15,16,17].

## 5. Limitations

The low number of patients included in this study, the single-centre setting, and the retrospective design may all impact the results. Relying solely on electronic health records may have limited the generalisability of the findings. Additionally, we did not have direct access to paramedics’ documentation.

## 6. Conclusions

Our study shows that there is still a need to include analgesia and sedation in peri-resuscitation care protocols, as only two-thirds of the patients received sedation and only half received analgesics.

## 7. Perspectives

Further research is needed to optimise pain and sedation management strategies during and after CPR. With some evidence that therapeutic hypothermia should probably be limited to a specific, narrow group of patients [18], there is renewed interest in pharmacological means of brain protection. Investigating the potential benefits of drugs such as ketamine, which has unique analgesic and sedative properties, could provide new insights into improving patient care in emergency settings [19].

## Figures and Tables

**Table 1:** Demographic and clinical characteristics of the CPR cohort (*n* = 131).

Variable	Total
Sex	
Male	74 (56.5)
Female	57 (43.5)
Causes of SCA as primary diagnoses at discharge from the emergency department (%)	
• Heart failure	40 (30.5)
• Myocardial infarction	25 (19.1)
• Cardiac arrhythmia	16 (12.2)
• Pneumonia	13 (9.9)
• Asphyxia	10 (7.6)
• Sepsis	6 (4.6)
• Haemorrhage	5 (3.8)
• Coronavirus disease (COVID-19)	5 (3.8)
• Pulmonary embolism	4 (3.0)
• Cardiac arrhythmia + asphyxia	1 (0.8)
• Cancer	1 (0.8)
• Other	5 (3.8)

Results presented as medians [lower and upper quartiles] or absolute numbers (percentages); SCA—sudden cardiac arrest; CPR—cardiopulmonary resuscitation.

**Table 2:** The incidence of chest wall injury in the CPR cohort (*n* = 126).

Variable	Total	Female	Male	*p*-Value
None	96 (76.2%)	48 (87.3%)	48 (67.6%)	0.036
Rib fracture	22 (17.5%)	5 (9.1%)	17 (23.9%)	
Rib + sternal fracture	8 (6.3%)	2 (3.6%)	6 (8.4%)	

Results are presented as absolute numbers (percentages); CPR—cardiopulmonary resuscitation.

**Table 3:** Sedative and analgesic drugs used during the peri-resuscitation period (*n* = 168).

Variable	Total
Midazolam	76 (45.2)
Propofol	35 (20.8)
Morphine	9 (5.3)
Fentanyl	45 (26.8)
Ketamine	2 (1.2)
Other	1 (0.6)

Results presented as absolute numbers (percentages).

## Data Availability

The data that support the findings of this study are available from the main author (S.D) and L.T. upon reasonable request.
